# Caries and Necrosis of the Jaws

**Published:** 1886-12

**Authors:** J. H. Martindale

**Affiliations:** Minneapolis, Minn.


### CARIES AND NECROSIS OF THE JAWS.

BY J. H. MARTINDALE, M. D., D. D. S., MINNEAPOLIS, MINN.

READ BEFORE THE MINNESOTA STATE DENTAL SOCIETY.

The death and ultimate separation of portions of the margins of the maxillæ are not of uncommon occurrence. The thin edge of alveolus may exfoliate after an extraction, no matter how skillfully the operation may have been performed, and its results may be confined alone to a slight prolongation of the period of healing. But in the stead of this very limited death of bone, the disease may be much more extensive and involve very large portions, or the whole, of either jaw.

The causes that bring about necrosis and caries of the jaws are various, and cannot always be traced. Among them are the exanthematous diseases of children (measles, scarlet fever, chicken pox, scarlatina, etc.). In these cases the sequestrum is generally limited to the sockets of one or two temporary teeth, very often carrying away with it the follicles of the permanent ones, this being, probably, a by no means infrequent cause of the failure of these teeth to appear, a condition of things frequently presenting in our dental practice.

Constitutional syphilis is a predisposing condition to necrosis, and such an instance as that cited by Sir Christopher Heath, in his work on Diseases of the Jaws, where a dentist had suit brought against him for a case of necrosis ensuing in a jaw after the extraction of a tooth, the patient being a syphilite, is not uncommon.

Blows, kicks, bruises, and the like, are frequent causes of caries and necrosis, especially in individuals of scrofulous or anæmic habit. A great many, undoubtedly the larger number, of diseased conditions of the maxillæ, originate in diseases of the teeth, and in unfortunate, or even careless dental operations. Among the former causes may be enumerated devitalized pulps, encysted teeth (or odontocelles), and delayed eruption of the *dens sapientiæ,* and among the latter, heavy malleting, roots improperly filled, and roots filled with improper material (as cotton). I have also even seen in my own practice, I am sorry to say, necrosis with the exfoliation of alveolar process, as a result of application of arsenious acid to a pulp for the purpose of devitalization. I am persuaded that this, not infrequently, occurs in cases where the devitalizing agent was used with every protective care, as in my own, where the amount of arsenious acid was very slight, and only applied to the pulp after the tooth containing the pulp had been thoroughly covered by the rubber dam, and over it an oxy-chloride of zinc filling.

Another mineral poison, with specific tendencies to effect necrosis of the jaws, is phosphorus, the fumes of which, inhaled by workmen in match factories, induce the most extensive necrosis. But we shall not deal with phosphor-necrosis in this paper, as it is of very infrequent occurrence in this part of the country.

To the relief of the dental surgeon be it stated, that in addition to those cases of diseased maxillæ, the unquestioned result of cachexia, exanthema, mineral poisons and traumatisms, many cases occur which are, without doubt, idiopathic. Heath says (Vol. 2, page 459): "I have seen the disease (necrosis of the jaws) occur in otherwise healthy persons without any assignable cause. In this way I have seen the whole of the alveolar process of the upper jaw exfoliate in a young lady, otherwise perfectly healthy, and I have several times had occasion to remove large portions of the lower jaw, in one case more than half of the bone, for necrosis that could not be referred to any assignable cause." And again, Tomes in his System of Dental Surgery (page 496), after describing a case of necrosis of the incisive region of the upper jaw, where a large portion of the alveolus in that region was involved, and sufficient of the maxillary bone proper to have caused a large opening into the nares, concludes by saying: "The most remarkable feature in the case was the entire absence of any assignable cause of mischief. The patient was a healthy man of middle age, with no history of syphilis; the tooth had been only slightly decayed, and had been successfully filled with gold, years previously. No blow had been received; in fact, nothing whatever could be discovered to account for the lesion."

Stanley, also, in his work on "Diseases of the Bones" (page 71), speaks as follows: "A large portion of the lower jaw in young persons occasionally perishes without any previous derangement of the health, local injury, or other apparent cause. But in some cases, an aching of the bone has preceded the death of it. Such cases of necrosis usually occur in early life, between the fourth and twentieth years, but rarely later."

Authorities in evidence of the frequency of these cases of idiopathic origin of maxillary necrosis might be quoted in large number. I lay stress upon this fact, in consequence of the too frequent reproach cast upon careful and discreet dental practitioners, on the score of their ill-advised or rough operations having induced the disease. I believe that, in certain cases, the tissues adjacent to the field of a dental operation are in a condition peculiarly prone to take upon themselves an inflammation, which, becoming a pronounced ostitis, then breaks down into a veritable necrosis or caries; this, too, without any cachexia, which should naturally be supposed to predispose to this result. None the less, it should be constantly borne in mind that, by improper filling of the pulp canals, excessive and violent wedging, heavy malleting and the like, many cases of caries and necrosis are induced.

Diagnosis: In the outset, the indications that attend necrosis are not to be distinguished from alveolar periostosis; but soon, instead of confining itself to local or circumscribed swelling, the gums become congested and swollen over a considerable area, and pus oozes from the margins of the gums, the teeth become loose, and in a few weeks the alveolus becomes loose and lies in its position bathed in pus. Pain is common to the early part of the disease, and is usually supposed to be toothache; the patient's health frequently suffers greatly, all the symptoms of septic fever, high temperature, rigors, sweats, etc., being frequently present, and more than in necrosis generally is maxillary necrosis fraught with special danger to the health, in consequence of the large amount of the pus and sanious products which are often voided into the oral cavity and swallowed. A very common condition encountered in our practice (I have hesitation in including it in the scope of either maxillary caries or necrosis, yet it is very generally regarded by practitioners as at least strongly allied to an ulcerative or necrotic disease of the maxillæ) is such as, for example's sake only, may be briefly outlined in the following as a type.

A patient goes through the history of the origin, symptoms and natural suppurative termination of what is generally known as an alveolar abscess, finally voiding its contents by a fistula through the gums adjacent to the point of origin of the disease; one or more teeth in the meantime are found to have become quite loose; a probe passed into the fistula soon plunges into a large cavern or cyst excavated out of the substance of the alveolus, and sometimes of the maxillary bone proper. This excavation will be found to have protruding into it the root or roots of one or several teeth, and the educated and discerning touch of one accustomed to discriminating with the probe alone, will readily detect the presence of diseased and spongy bone forming the walls of the cavity, and often, also, the roots of the teeth protruding into the cavity will be found to be themselves partially or wholly absorbed by the diseased condition.

Such a case as I have outlined above is a condition of very common occurrence in dental practice. Having voided their contents through a fistula. they often succumb to the simplest but radical means applied by tapping and cleaning root canals, etc.; or they persist for months, even years, in a purulent discharge through the alveolus, which is either persistent, or intermittent for quite a space of time. I shall have cases in my own practice to cite in this paper, in evidence of the treatment of this very common disease of the alveolus. Although unaccompanied by the systemic disturbances common to an equally large area of lost bone in other parts of the body, and although unaccompanied, as a rule, by distinct and considerable sequestra, I think the scope of this paper should include them.

Treatment: All loose roots that cannot be made useful by the attachment to them of artificial crowns should be extracted. If, upon careful examination with a probe, we find that the bone is not diseased very extensively, that is, if caries has not extended immediately surrounding the roots of one or two teeth, absorption and extrusion of dead bone may usually be effected by the use of sulphuric acid brought directly in contact with the area of diseased bone. The preparation of the acid that I prefer is that known as the "Aromatic Sulphuric Acid" (a thirteen and a half per cent. solution of the sulphuric acid of commerce, together with the aromatic principles of various spices). My method of using it is to inject it through the fistulous opening and directly in contact with the diseased bone by means of a hypodermic syringe, provided with a blunt-ended needle, keeping it there for several minutes. I commence with a strength of about 70 per cent. solution of the aromatic sulphuric acid, and soon increase it to full strength. This treatment, conjoined with the use of injections of the peroxide of hydrogen, the patient's health at the same time being toned up with tonics, and the bowels kept freely open by the use of saline waters and aperients, will often effectually remedy the trouble. But in some instances the diseased bone is not gradually consumed and cast off, but either large sequestra are exfoliated, needing surgical assistance in their removal, or a considerable area of honeycombed carious bone is formed, needing removal in like manner by other means than by the dissolving properties of the acid. My treatment of such conditions will be set forth more in detail in the recital of a few cases from my own practice. It should be remembered that the termination of a periostitis in a carious or necrotic condition may often be averted by freely and deeply scarifying the gums, use of saline cathartics, rest, attention to diet, tonics, etc., if commenced early enough.

### CASES.

CASE I. In February of 1882, a young boy of nineteen years of age was brought to me by his father for a condition of fixation of the jaw, accompanied by a large swelling of the right cheek, and enlargement of the lymphatics in the cervical and sub-maxillary region. The circumstance of extensive cicatrices upon the face, resulting in consequent contraction of the facial muscles and distorted features, immediately induced me to ask of his father the cause, and I was informed that the boy was affected seriously with scrofula, and that these were the result of several running sores of the face.

As the abscess (for such it was) upon his face was already pointed, and I could not get into his mouth to void its contents there, I lanced externally, and very fœtid, greenish-yellow pus was copiously poured forth. Upon closely examining this pus, I found therein contained small spiculæ of bone, and upon exploring the abscess tract with a silver probe I was easily able to discern the presence of an opening in the inferior maxillary bone, just before its junction with the ascending ramus. There seemed to be no large sequestra of bone detected by the probe, but rather a honeycombed or porous feel to the walls of the cavity. The abscess cavity was then very thoroughly irrigated with carbolized water, and the boy was sent home with instructions to take a tonic of iron and quinia, good nutritious food, and return in two days. It should be remarked that the patient had been having high evening temperatures for several days, and his general condition was much run down by pus absorption.

Upon his appearance at my office in two days, I found my patient with the abscess still discharging (I had placed a seton in the opening), but the enlarged condition of his face very much decreased. I was now able, by the aid of a wedge and anæsthesia, to get his mouth open, and the evidence of the origin of his trouble (combined with the scrofulous cachexia) was soon evident; a late, and difficult erupting, *dens sapientiæ.* Said tooth was lodged, with its masticating surface presented in apposition to the posterior approximal surface of the second molar in front of it, and only one cusp emerging above the gums, which were very turgid and inflamed. With a physic forceps I managed to extract the offending tooth, and was then able to run my probe into the socket, and from there to the diseased tract in the maxillary bone, and so on out through the opening in the face which I had previously made.

I now had what I wanted, "through" drainage, and it was easy for me to inject my carbolized or other fluids (I had not then peroxide of hydrogen at my command) through the whole diseased tract. My treatment now was a thorough scraping of the walls of the tract with a dull scraping instrument, thorough and frequent irrigation with antiseptic washes, and a tonic treatment of the system with good food, iron, wine and quinia. After five or six days, the pus ceased to run, and in three or four days more the external wound was allowed to heal, the boy's health was renewed, and the case dismissed.

CASE II. I was called by a dentist, Dr. X., to see a case that had developed an undoubted carious or necrotic character. The patient was a lady, aged about thirty-five years, married, and reported herself as possessing usually good health. Upon examination of her mouth, it was seen that pus was escaping copiously from the gums surrounding the superior left central, lateral, canine and first bicuspid. As to its cause no sufficient origin could be assigned; it had supervened not very long after the insertion of a not very large approximal filling in the lateral, but the circumstance of there being no conditions apparent at the time of the operation to contra-indicate any operation, together with the fact of its smallness and simplicity, seemed to preclude the probability of the filling being the direct cause; the case seemed to point rather to being one of those previously alluded to in this paper, whose origin can be assigned to no direct source. For five or six weeks she had been subjected to the customary and proper treatment with the hypodermic injection of aromatic sulphuric acid, the disease seeming not to abate, but rather to grow worse and worse. The patient's health also had begun to suffer materially, her temperature running to 104, and she was generally weak and run down. Acid treatment having failed, it was a question as to what should next be done. Upon examining the parts with a probe, it was easy to determine the presence of much diseased bone, and several detached pieces of the central, lateral and canine were so loose that it seemed as though it would be easy to remove them with the fingers, while the first bicuspid had begun to be seriously affected. After careful examination it was deemed best to operate for removal of the dead bone, which was done as follows: The patient anæsthetized, holes were drilled into the pulp cavities from the lingual aspects of the central, lateral and canine teeth, all of which were found to contain dead pulps. Then a splint which we had made of vulcanite was placed in the mouth, and to it the central, lateral and canine were securely ligated. This was to prevent the dropping out of these teeth during the operation, which would otherwise undoubtedly have occurred. I then made a linear incision about an inch and a half in length, parallel with the margin of the gum, and about a third of an inch above the margin; in then passing down into the gum the alveolus was found to be entirely dead, some of it absorbed and carried off with the pus, and a number of the dead pieces were lying detached in the wound. These were removed with gouges, chisels and spicula forceps, the rest of the dead bone involving, we found as we went on, a considerable portion of the anterior external face of the maxillary bone. When the bone had all been removed, it was found that we had a space presenting an aperture about an inch long and three-fourths of an inch high, and perhaps nearly an inch deep. It extended posteriorly to the antrum, and in front to the median line; the palatal surface or floor of our cavity was all devoid of bone, and naught remained there for quite a space save a mucus membrane partition separating us from the mouth. In one place the cavity opened into the nasal cavity, about an inch back from the nostrils. The roots of the lateral and canine teeth projected, entirely bare of any bony investment, into this cavity, as also did the apices and sides of the bicuspid and central, which were approximal to the diseased tract. During our operation the canine came out entirely, and although we began to think that it was a very forlorn chance to save any or all of the teeth, we tied it back in its place to see if perchance new tissue would close around it.

Every step in the operation, it should be noticed, was done under constant injection of bichloride of mercury water, and all the probes and instruments were thoroughly immersed in this antiseptic wash before and during use. We now plugged up the opening with three pieces of cheese cloth charged with iodoform, each one-half inch wide and four inches long (they came just level with the top of the wound), and then left the patient until the next day. Considerable swelling of the face was then found, but otherwise everything seemed favorable. Upon removing the dressing we found, to our satisfaction, not a trace of pus, but rather healthy lymph, and every evidence of a healthy wound. Carbolized water was injected freely, and it was found that considerable of the injection passed into the nose through the opening that extended into it. To summarize in brief the issue of the case, I will say, that from the day of the operation, we saw no pus; when about every five days we removed the dressing, we found the reparative process going on splendidly from the bottom; soon the opening into the nares closed, tissue formed around the bicuspid and central teeth, and at the end of about six weeks from the time of the operation, osseous tissue had been formed everywhere, save on top of the roots of the incisor and lateral. At the present writing, it seems as though the canine would be lost; the other teeth will, however, be profitably saved, and even though none of the teeth involved had been saved, the interest attaching to the loss and repair of so large a portion of the alveolus and maxilla has been deemed sufficient excuse for so detailed an account. The patient's general health, which is now perfect, began to amend from the time of three or four days after the operation.

The next and last case with which I shall burden you is one of very frequent occurrence in our practices; not usually very dangerous to the general health, it is true, but often attended with very considerable loss of alveolar substance and loosening of the teeth. The disease is sometimes very obstinate in yielding to local medication, or to the use of aromatic sulphuric acid, but by simple surgical removal of the diseased bone it will almost always cease. To illustrate, I will select at random from a number of similar cases in my practice.

Case III. Miss M—— applied to me to relieve her of a discharge issuing from a fistulous opening in the gums, between the left lateral and canine superior teeth. She reported the discharge to have existed almost continuously for over a year. Upon examination of the lateral, which was filled upon its mesial approximal surface with gold, I easily discovered it to contain a dead pulp. I immediately drilled a hole through the lingual aspect of the tooth, and, upon entering the pulp cavity, found it to be filled with a fluffy substance which, upon removal, was found to be a mass of cotton wool, which the patient said had been placed there about two years ago by a dentist who had filled the tooth. I then, after as thoroughly cleansing the pulp canal as possible, filled the said canal with a dressing of cotton floss and iodoform, and dismissed my patient for a week. When next she came I removed the iodoform dressing, thoroughly washed out the canal, and entirely filled it with oxy-chloride of zinc. I then took a probe, and passing it down through the fistulous opening was able easily to discern the presence of soft, dead bone, which I proceeded to remove with a bur in the dental engine; first, however, to reduce pain and diminish hemorrhage, I injected as deep as I could penetrate with a blunt hypodermic needle into the fistula, about 8 m. of a four per cent. aqueous solution of hydrochlorate of cocaine. I then proceeded in two or three minutes to remove with my bur the dead bone, which I could easily tell by its feel, and entered a cyst, or enclosure, about the size of a small marble, to which the fistula led. The sides of this I thoroughly scraped until I was sure the walls were composed entirely of live bone, and also burred off the top of the lateral tooth which was projecting into the wound. I then very thoroughly washed out the cavity with carbolized water, and after inserting in the orifice of the wound a tent of cotton I dismissed the patient, who said she had suffered during the operation no pain. The next day I found no pus, and injected freely with peroxide of hydrogen, and giving her a syringe and some peroxide directed her to use it herself three times a day. She did so; in three or four days the pus ceased to flow, granulations of new tissue began to appear, and in about four weeks the orifice healed up, and during ten months past has shown no disposition to recur.

